# The Vesicle Priming Factor CAPS Functions as a Homodimer via C2 Domain Interactions to Promote Regulated Vesicle Exocytosis*

**DOI:** 10.1074/jbc.M116.728097

**Published:** 2016-08-15

**Authors:** Matt Petrie, Joseph Esquibel, Greg Kabachinski, Stephanie Maciuba, Hirohide Takahashi, J. Michael Edwardson, Thomas F. J. Martin

**Affiliations:** From the ‡ Department of Biochemistry,; ¶ Program of Molecular and Cellular Pharmacology, and; § Integrated Program in Biochemistry, University of Wisconsin, Madison, Wisconsin 53706, and; the ‖ Department of Pharmacology, University of Cambridge, Cambridge CB2 1PD, United Kingdom

**Keywords:** dimerization, exocytosis, inositol phospholipid, SNARE proteins, vesicles

## Abstract

Neurotransmitters and peptide hormones are secreted by regulated vesicle exocytosis. CAPS (also known as CADPS) is a 145-kDa cytosolic and peripheral membrane protein required for vesicle docking and priming steps that precede Ca^2+^-triggered vesicle exocytosis. CAPS binds phosphatidylinositol 4,5-bisphosphate (PI(4,5)P_2_) and SNARE proteins and is proposed to promote SNARE protein complex assembly for vesicle docking and priming. We characterized purified soluble CAPS as mainly monomer in equilibrium with small amounts of dimer. However, the active form of CAPS bound to PC12 cell membranes or to liposomes containing PI(4,5)P_2_ and Q-SNARE proteins was mainly dimer. CAPS dimer formation required its C2 domain based on mutation or deletion studies. Moreover, C2 domain mutations or deletions resulted in a loss of CAPS function in regulated vesicle exocytosis, indicating that dimerization is essential for CAPS function. Comparison of the CAPS C2 domain to a structurally defined Munc13-1 C2A domain dimer revealed conserved residues involved in CAPS dimerization. We conclude that CAPS functions as a C2 domain-mediated dimer in regulated vesicle exocytosis. The unique tandem C2-PH domain of CAPS may serve as a PI(4,5)P_2_-triggered switch for dimerization. CAPS dimerization may be coupled to oligomeric SNARE complex assembly for vesicle docking and priming.

## Introduction

In regulated exocytosis, vesicles fuse with the plasma membrane in response to Ca^2+^ elevations, releasing cargo such as peptide hormones or neurotransmitters to the extracellular space. CAPS^3^ (also known as CAPS-1, CADPS, and Unc-31p) was discovered by its activity in promoting Ca^2+^-triggered dense core vesicle exocytosis in permeable neuroendocrine cells (1, 2). Subsequent studies demonstrated the essential role of CAPS in the docking and priming of dense core vesicles and synaptic vesicles in endocrine and neural cells (3–14). CAPS proteins contain at least three important functional domains corresponding to the C2 domain, pleckstrin homology (PH) domain, and Munc13 homology domain 1 (MHD1) (Fig. 6*A*) (15). Based on sequence homology in C-terminal regions, CAPS is a member of a CATCHR (complexes associated with tethering containing helical rods) family of tethering/priming proteins that operate at various stages in the secretory pathway (16–18). Munc13-1/2 proteins in this family function similarly to CAPS but non-redundantly at priming steps in vesicle exocytosis (3, 7, 12, 19, 21).

The three annotated CAPS domains are important for activity in regulated vesicle exocytosis. The C2 domain of CAPS is essential for nervous system function in *Caenorhabditis elegans* indicated by the uncoordinated phenotype of *unc-31(e714*) *and unc-31(ox299*) strains that harbor point mutations in the C2 domain of the conserved worm CAPS ortholog UNC-31p (22). The exact role of the C2 domain in CAPS function was unknown and is revealed in the current study. The adjacent PH domain of CAPS has been characterized as a PI(4,5)P_2_-binding domain required for CAPS activity in supporting regulated exocytosis in cells and for enabling PI(4,5)P_2_- and SNARE-dependent liposome fusion (4, 19, 23–25). The PH and C2 domains of CAPS are closely adjacent (Fig. 6*A*) and could exhibit mutual regulation as in other tandem domain proteins (26). Last, CAPS interacts with Q-SNAREs (syntaxin-1 and SNAP-25) and, at lower affinity, with the R-SNARE (VAMP2) that comprise the neuronal SNARE complex (27). SNARE binding by CAPS is mediated by MHD1 or more C-terminal domains within the CATCHR region (15, 28). Current models for function in vesicle exocytosis suggest that CAPS on dense core vesicles (3) engages PI(4,5)P_2_ and Q-SNAREs on the plasma membrane via its PH and MHD1 domains, respectively, to promote SNARE complex assembly for vesicle docking and priming (17, 29).

CAPS promotes the formation of dimers of SNARE complexes on liposomes, but it was unclear whether this was mediated by the dimerization of CAPS (23, 30). Early hydrodynamic studies of partially purified protein suggested that CAPS was a dimer (2). However, three protein bands were identified in native gel electrophoresis of purified CAPS, but the oligomerization state of these CAPS forms was not established (31). Here we report that purified soluble recombinant CAPS is in monomer-dimer equilibrium favoring the monomer but that the membrane-bound active form of CAPS is a dimer. PI(4,5)P_2_ and Q-SNARE interactions stabilize the CAPS dimer. The role of the CAPS C2 domain in mediating homodimerization was revealed by studies of the mammalian counterparts of *unc-31* C2 domain mutants (22) and a C2 domain deletion that exhibit altered dimer formation and loss of function in vesicle exocytosis. Last, analysis suggests that CAPS dimerizes similarly to Munc13-1/2 using conserved homodimerization residues in its C2 domain.

## Results

### 

#### 

##### Biochemical Characterization of CAPS Oligomerization

**FIGURE 1.**
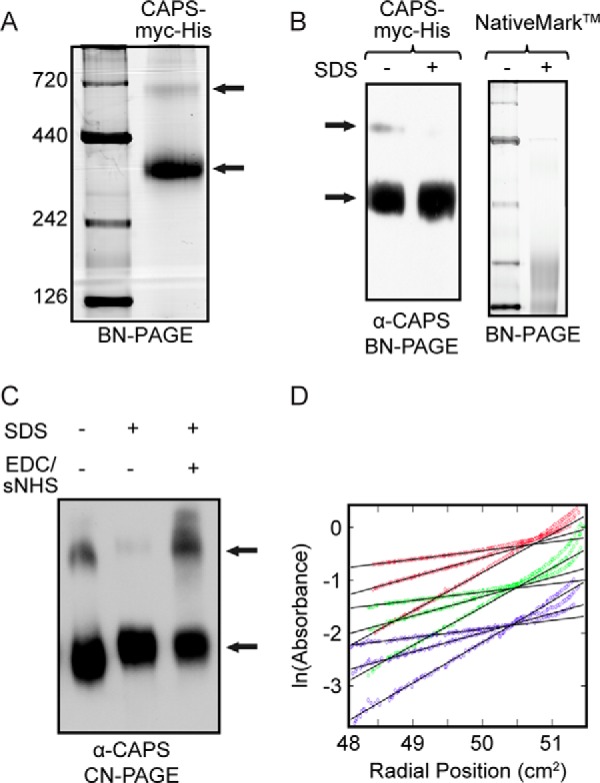
**Soluble CAPS is predominantly monomeric.** *A*, BN-PAGE (blue native gel, 6–14%) of NativeMark^TM^ protein ladder and purified recombinant CAPS-Myc-His, representative of eight similar analyses. *B*, Western blot (α-CAPS) from BN-PAGE of purified CAPS-Myc-His without or with 2% SDS (*left*) and NativeMark^TM^ protein ladder without or with 2% SDS (*right*), representative of three similar analyses. *C*, Western blot (α-CAPS) of purified CAPS-Myc-His from CN-PAGE (clear native gel) without or with 2% SDS and with 2% SDS following CAPS cross-linking with 0.5 mm 1-ethyl-3-(3-dimethylaminopropyl) carbodiimide and 1.25 mm sulfo-NHS for 2 h at room temperature, representative of two similar studies. *Arrows*, high mobility CAPS-Myc-His monomer and low mobility CAPS-Myc-His oligomer. *D*, sedimentation equilibrium data from analytical ultracentrifugation of CAPS. Raw data (*squares*) are shown for three different speeds (3600 (*blue*), 6000 (*green*), and 8800 (*red*) rpm) with three different loading concentrations for each speed. Fits (*black lines*) were derived from a non-interconverting two-species model where the observed molecular weight/monomer molecular weight for each species is ∼1.0 and ∼14, corresponding to monomer and higher molecular weight unresolved components, respectively.

We reported that native CAPS is a dimer, but this analysis was conducted with partially purified protein using indirect hydrodynamic criteria (2). However, a reassessment of oligomerization with a highly purified active recombinant CAPS-Myc-His protein (32) indicated that the protein migrated in blue native gel electrophoresis as a major 350-kDa and a minor 700 kDa band (Fig. 1*A*). Treatment of the protein with SDS, which was effective at disassembling oligomeric molecular mass standards, collapsed the 700-kDa but not the 350-kDa form of the protein (Fig. 1*B*). This suggested that the 350 kDa band corresponds to a monomer, whereas the 700-kDa band is an oligomer. Consistent with this, zero length cross-linking partially converted the CAPS-Myc-His monomer to an oligomer that resisted SDS-induced disassembly (Fig. 1*C*). The minor amounts (∼6%) of oligomer in CAPS-Myc-His (*n* = 27) did not differ significantly from that detected in native cytosolic CAPS (*n* = 11), indicating that epitope tags were not responsible for oligomer formation (*e.g.* see Fig. 4*C*). The molecular mass of CAPS-Myc-His on SDS-PAGE is ∼145 kDa, which indicates that the 350-kDa CAPS-Myc-His form in blue native gels migrates as a non-globular protein with an oblate shape. To assess its native molecular mass independent of shape, purified CAPS-Myc-His was analyzed by analytical equilibrium ultracentrifugation, which showed that the major species of soluble CAPS-Myc-His is a ∼150-kDa monomer (Fig. 1*D*). Minor amounts of a higher molecular weight oligomer(s) were not well resolved by this method.

**FIGURE 2.**
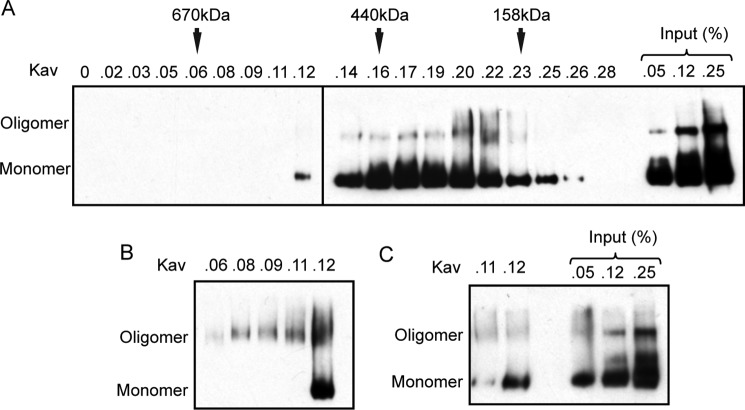
**Oligomeric forms of CAPS are in dynamic equilibrium.** *A*, gel filtration of CAPS-Myc-His detected by Western blot of CN-PAGE. Input to the column is indicated. The column was calibrated by the elution of aldolase, ferritin, and thyroglobulin. 0.16–0.19 *K*_av_ fractions correspond to CAPS monomer. *B*, longer exposure of Western blot of 0.06–0.12 *K*_av_ fractions to detect oligomer. Gel analysis was conducted immediately after gel filtration. *C*, Western blot of 0.11 and 0.12 *K*_av_ fractions kept on ice for 24 h after gel filtration. Purified oligomer partially dissociated to monomer. Results shown are representative of two replicate studies.

Gel filtration studies indicated that the dominant form of CAPS-Myc-His migrates with a Stokes radius (*R_S_*) of ∼59 Å similar to a 350-kDa globular protein (Fig. 2*A*). This is consistent with a moderately elongated 145-kDa monomer with an *R_S_*/*R*_min_ value of ∼1.7 (33). The 0.17 *K*_av_ monomer fractions from gel filtration analyzed by native gels contained ratios of monomer to oligomer similar to the input, indicating that gel-filtered monomer partially converts to oligomer (Fig. 2*A*). Long exposure of the Western blot revealed oligomer in the 0.06–0.12 *K*_av_ fractions (Fig. 2*B*), but analysis of these fractions 1 day later showed substantial conversion to monomer (Fig. 2*C*). We conclude that soluble CAPS-Myc-His is in dynamic monomer-oligomer equilibrium favoring monomer.

##### Atomic Force and Single Molecule Microscopy

**FIGURE 3.**
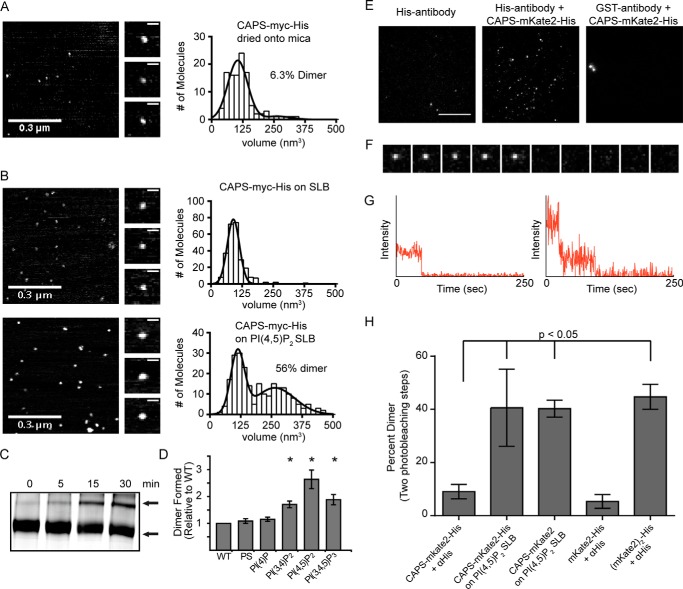
**CAPS oligomer is a dimer.** *A*, atomic force microscopy volume analysis of purified CAPS dried onto mica (*n* = 108). Representative images of AFM field (*left*), representative images of single CAPS-Myc-His molecules (*middle*), and histograms of the CAPS-Myc-His volume distribution (*right*) are shown. Volume data were fit to a double Gaussian with volumes of 109 nm^3^ for monomer and 264 nm^3^ for dimer. *B*, atomic force microscopy analysis of purified CAPS-Myc-His added to SLB (*top*, *n* = 448) or SLB containing PI(4,5)P_2_ (*bottom*, *n* = 238) detected monomers at 94 nm^3^ or monomers at 115 nm^3^ plus dimer at 261 nm^3^, respectively. Inclusion of PI(4,5)P_2_ in SLBs significantly increased the amount of CAPS-Myc-His dimer (*p* < 0.0001, Mann-Whitney-Wilcox test). *C*, CAPS-Myc-His dimers increased upon PI(4,5)P_2_ addition as indicated by BN-PAGE of 5 μg of CAPS-Myc-His incubated with 1.5 μg of PI(4,5)P_2_ for 0–30 min. *Arrows*, high mobility monomer and low mobility dimer. Results shown are representative of six experiments. *D*, incubations and analysis similar to *C* were conducted with the indicated phospholipids showing that CAPS-Myc-His dimers were preferentially induced (-fold increase is shown) by PI(4,5)P_2_ (mean ± S.D. (*error bars*); *, *p* < 0.001, *n* = 5). *E*, pull-down of single CAPS-mKate2-His molecules. TIRF microscopy images showed puncta when CAPS-mKate2-His was retained by His antibody (*middle*) but not with control antibody (*right*). *Scale bar*, 10 μm. *F*, TIRF images (collected at 2 Hz) showing a one-step photobleach event for immobilized CAPS-mKate2-His. Image size is 2.5 × 2.5 μm. *G*, representative fluorescence intensity *versus* time analysis for 1-step and 2-step photobleach events for CAPS-mKate2-His. *H*, percentages of puncta exhibiting two photobleaching steps were quantified for CAPS-mKate2-His tethered by His antibodies (*n* = 486), CAPS-mKate2-His bound to PI(4,5)P_2_ SLBs (*n* = 296), CAPS-mKate2 (lacking His tag) bound to PI(4,5)P_2_ SLBs (*n* = 209), and mKate2-His (*n* = 307) or (mKate2)_2_-His (*n* = 117) tethered by His antibodies. Note that the (mKate2)_2_-His dimer was only 50% pure and contaminated with fluorescent monomeric mKate2. Values are mean ± S.D.

To further assess the oligomerization state of CAPS, we employed atomic force microscopy (AFM) and single-molecule microscopy. AFM measures protein volume and can be used to determine oligomer stoichiometry (34). The volume of CAPS-Myc-His was measured after drying directly onto mica or after it was added to a supported lipid bilayer (SLB) on mica with or without PI(4,5)P_2_. The majority of CAPS-Myc-His on mica (Fig. 3*A*) or on the SLB lacking PI(4,5)P_2_ (Fig. 3*B*, *top*) was monomer. However, the inclusion of PI(4,5)P_2_ in SLBs strikingly enhanced the amount of CAPS-Myc-His present as a dimer (Fig. 3*B*, *bottom*). The stimulatory effect of PI(4,5)P_2_ on CAPS-Myc-His oligomerization was confirmed in native gel electrophoresis studies showing that CAPS-Myc-His oligomerized upon incubation with PI(4,5)P_2_ micelles (Fig. 3*C*). PI(4,5)P_2_ was the most effective phosphoinositide for inducing oligomerization (Fig. 3*D*). The results indicate that CAPS-Myc-His exists in two states and that PI(4,5)P_2_ binding promotes the formation of oligomers that correspond to dimers.

Single molecule microscopy can be used to determine oligomeric state by monitoring the number of photobleaching steps for fluorescently tagged proteins (35). CAPS-mKate2-His molecules were held by His antibodies (Fig. 3*E*), and photobleaching steps were monitored by total internal reflectance fluorescence (TIRF) microscopy (Fig. 3, *F* and *G*). As controls, monomeric mKate2 was shown to exhibit one-step bleaching, whereas dimeric mKate2 exhibited an increased number of two-step bleaches (Fig. 3*H*). CAPS-mKate2-His mainly exhibited 1-step bleaching, but ∼5% of the molecules exhibited two-step bleaching, indicating that a minor population of dimers is present (Fig. 3*H*). The inclusion of PI(4,5)P_2_ markedly enhanced dimerization of the CAPS-mKate2-His protein (Fig. 3*H*). To exclude the role of the His tag, we prepared a CAPS-mKate2 protein, which exhibited similar PI(4,5)P_2_-enhanced dimerization (Fig. 3*H*). Collectively, the AFM and single molecule studies indicate that CAPS oligomers correspond to dimers and that PI(4,5)P_2_ enhances CAPS dimerization.

##### CAPS Dimers Are Functional in Vesicle Exocytosis

**FIGURE 4.**
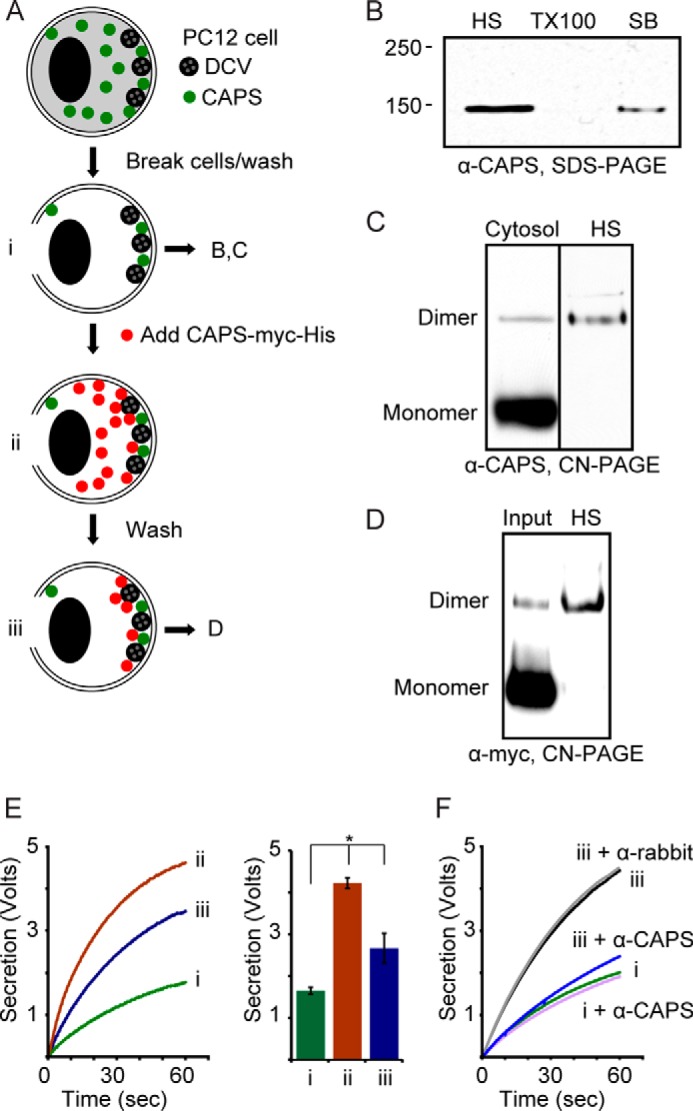
**CAPS dimer is the functional form of CAPS.** *A*, PC12 cells were passed through a ball homogenizer (10-μm clearance) to tear the plasma membrane and were washed in KGlu/BSA to remove cytosol (i). Purified CAPS-Myc-His was added to 20 nm (ii), and cell ghosts were washed with KGlu/BSA to remove unbound CAPS-Myc-His (iii). These three states were assessed for activity in *E. B*, washed cell ghosts (*i*) were sequentially extracted with 300 mm NaCl (high salt (*HS*)), 1% Triton X-100 (*TX100*), and SDS sample buffer (*SB*) with boiling. Samples were analyzed by Western blotting from SDS-PAGE with CAPS antibody. Results shown are representative of three studies. *C*, cytosol from PC12 cells (*left lane*) was compared with HS extract of endogenous CAPS from cell ghosts (*i*, *right lane*) in electrophoresis by CN-PAGE and Western blotting with CAPS antibody. Results shown are representative of four studies. *D*, purified CAPS-Myc-His (1% of input shown) was compared with 50% of HS extract from cell ghosts (*iii*) in electrophoresis by CN-PAGE and Western blotting with Myc antibody. Results shown are representative of two studies. Endogenous CAPS (*C*) or exogenous CAPS-Myc-His (*D*) retained by PC12 cell ghosts migrated as dimer. *E*, Ca^2+^-dependent norepinephrine secretion was monitored by rotating disk electrode voltammetry from cell ghosts indicated as *i*, *ii*, and *iii* in *A*. Ca^2+^ was injected at zero time. The results from three studies were averaged in the histogram (means ± S.E. (*error bars*)); *, *p* < 0.05. *F*, neutralizing CAPS antibodies inhibited the stimulation of secretion by prebound CAPS (*iii*). Rabbit IgGs (α-*rabbit*) were used as the control for CAPS antibodies (α-*CAPS*). Results shown are representative of two studies.

To determine the functional significance of the oligomeric states of CAPS, we assessed whether there was a preferential association of CAPS monomer or dimer with PC12 cell membranes. CAPS is a soluble cytosolic protein that partitions onto membranes (3, 25, 36). PC12 cells were mechanically permeabilized and washed to remove soluble contents (Fig. 4*A* (*i*)). Extensively washed permeable cells retain small amounts of bound native CAPS (4) that is efficiently extracted by a high salt wash (Fig. 4*B*) or by direct electrophoresis into native gels (not shown). In contrast to native cytosolic CAPS, which was ∼5% dimer, the extracted membrane-bound CAPS was entirely dimer as analyzed on native gels (Fig. 4*C*). Similar studies were conducted by adding purified CAPS-Myc-His to the permeable cells (Fig. 4*A* (*ii*)). The addition of purified CAPS to permeable PC12 cells stimulates Ca^2+^-dependent dense core vesicle exocytosis (4, 31). We tested the activity of the added purified CAPS-Myc-His and compared this with the activity of bound CAPS-Myc-His after a wash to remove unbound CAPS-Myc-His (Fig. 4*A* (*iii*)). CAPS-Myc-His bound to the permeable cells was exclusively dimer (Fig. 4*D*) and exhibited ∼50% of the activity (Fig. 4*E*, *curve iii*) of added CAPS-Myc-His (Fig. 4*E*, *curve ii*) in promoting Ca^2+^-dependent secretion. Control studies showed that the activity of bound CAPS-Myc-His was inhibited by neutralizing CAPS antibodies but not by control IgGs (Fig. 4*F*). Strikingly, the bound CAPS-Myc-His dimer represented only ∼1% of the input CAPS-Myc-His (Fig. 4*D*) but accounted for 40–60% of the detected activity (Fig. 4*E*). The results indicate that dimer is the active membrane-bound form of CAPS associated with Ca^2+^-triggered exocytosis.

**FIGURE 5.**
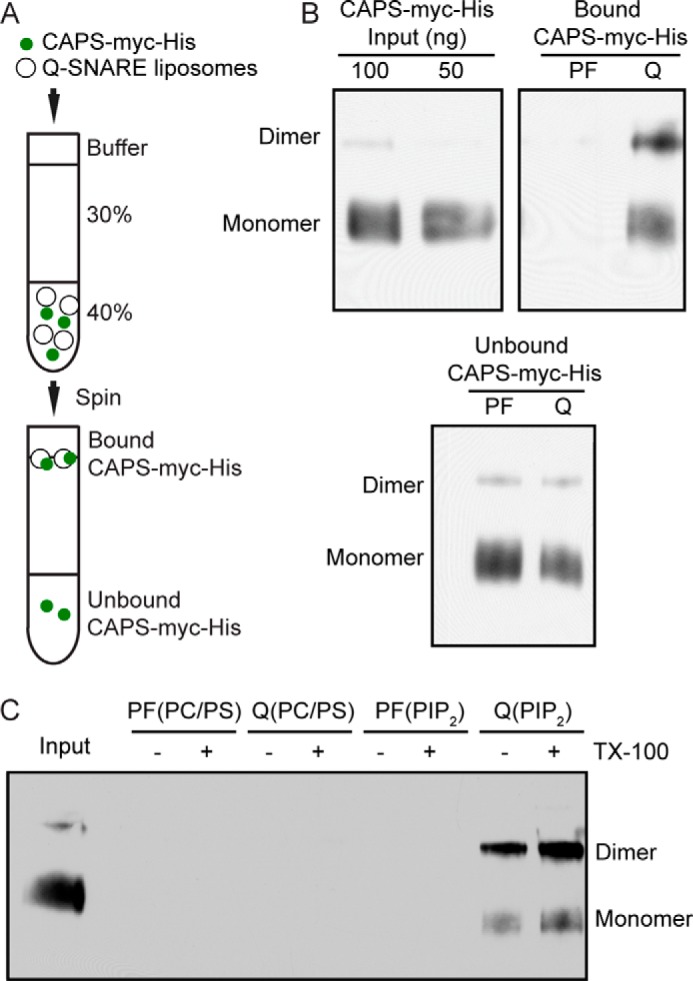
**CAPS dimers are enriched on liposomes containing Q-SNAREs and PI(4,5)P_2_.** *A*, diagram of flotation assay to separate bound from unbound CAPS-Myc-His in liposome binding studies. *B*, BN-PAGE Western blot of input (*left*), bound (*right*), and unbound (*bottom*) CAPS-Myc-His after flotation with either protein-free (*PF*) or PI(4,5)P_2_- and Q-SNARE-containing liposomes. Liposomes contained PC/PI(4,5)P_2_ (95:5). Results shown are representative of two studies. *C*, BN-PAGE Western blot of bound CAPS-Myc-His after flotation with either protein-free liposomes composed of PC/PS (85:15) or PC/PI(4,5)P_2_ (95:5) or Q-SNARE-containing liposomes with the same lipid compositions as indicated. Liposomes were also extracted into 1% Triton X-100 to ensure complete removal of bound CAPS-Myc-His. Results shown are representative of two studies.

Binding to SNARE proteins and PI(4,5)P_2_ is required for CAPS activity in exocytosis (15, 19), so we assessed the oligomeric state of purified CAPS bound to liposomes containing Q-SNAREs and PI(4,5)P_2_. The CAPS-Myc-His bound to Q-SNARE-, PI(4,5)P_2_-containing liposomes purified by flotation (Fig. 5*A*) exhibited increased dimer compared with input (Fig. 5, *B* and *C*). CAPS-Myc-His dimers were not evident in flotation studies with PC/PS-containing liposomes (Fig. 5, *B* and *C*) or with liposomes containing only PI(4,5)P_2_ (Fig. 5*C*). Although these results appeared to differ from the AFM studies with PI(4,5)P_2_-containing SLBs (Fig. 3*B*), we attribute this to the dissociation of CAPS-Myc-His during flotation when bound at low affinity to PI(4,5)P_2_-containing liposomes. The recovery of CAPS-Myc-His from liposomes containing only Q-SNAREs was also low (Fig. 5*C*) but corresponds to the synergy between PI(4,5)P_2_ and Q-SNAREs for CAPS activity on liposomes (23). Collectively, the results show that CAPS dimers preferentially associate with PI(4,5)P_2_ and Q-SNAREs and that membrane-bound dimers are the functionally active form of CAPS.

##### CAPS C2 Domain Mediates Dimerization

**FIGURE 6.**
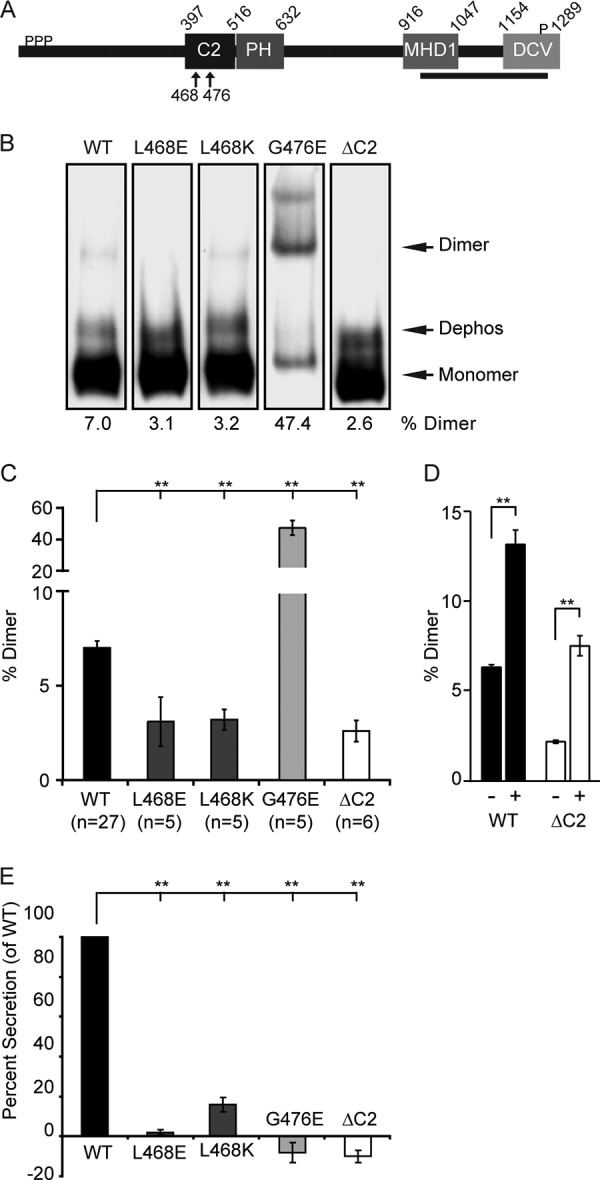
**Loss of function CAPS mutants exhibit altered dimerization.** *A*, domain architecture for *R. norvegicus* CAPS (NP_037351.1). C2 and PH domains were identified by multiple alignments in the SMART database. The MHD1 domain is from Ref. 25. The DCV binding domain is from Ref. 57. Ser phosphorylation (*P*) is from Ref. 31. CATCHR homology (*boldface underline*) is from Ref. 20. *B*, loss of function C2 domain mutants of CAPS exhibit abnormal dimerization. 100 ng of purified wild-type CAPS-Myc-His and mutants were analyzed by CN-PAGE and visualized by Western blot with Myc antibody. The CAPS-Myc-His band corresponding to dephosphorylated CAPS (*Dephos*) (31) was not routinely resolved from monomer in other CN-PAGE studies. *C*, the percentage of dimer for each protein indicated *below* each *lane* in *B* is plotted as histograms showing means ± S.E. (*error bars*) for the indicated *n* values. Comparisons with wild-type protein are indicated (**, *p* < 0.0001). *D*, wild-type CAPS-Myc-His and CAPS(ΔC2)-Myc-His were tested for dimerization in the absence (−) or presence (+) of PI(4,5)P_2_ as in Fig. 3*C* by BN-PAGE (**, *p* < 0.001; *n* = 3). *E*, activities of purified CAPS and mutants in a permeable cell secretion assay tested at 20 nm in triplicate (mean ± S.E.; **, *p* < 0.0001). Wild-type CAPS activity was set to 100%.

Additional evidence for the importance of dimerization in CAPS activity and for the role of the C2 domain in dimer formation was provided by studies of CAPS C2 domain mutants. Mutant proteins assessed were rat CAPS(G476E) and CAPS(L468K) plus variants (Fig. 6*A*) corresponding to the *unc-31(e714*) *and unc-31(ox299*) alleles in *C. elegans*, respectively (22). We also generated a C2 domain deletion protein CAPS(ΔC2). Mutant proteins were purified as Myc-His fusion proteins and analyzed by native gel electrophoresis to determine oligomerization state (Fig. 6, *B* and *C*), and in the permeable PC12 cell assay to determine activity (Fig. 6*E*). The alleles at Leu-468 (L468E and L468K) had significantly reduced dimer and exhibited loss of function in the permeable cell assay (Fig. 6, *B*, *C*, and *E*). The deletion construct CAPS(ΔC2) also exhibited reduced dimerization as well as reduced activity (Fig. 6, *B*, *C*, and *E*). We also tested the CAPS(ΔC2) protein with PI(4,5)P_2_ and found that PI(4,5)P_2_-induced dimerization was similarly attenuated (Fig. 6*D*).

Surprisingly, the CAPS(G476E) mutant exhibited strongly increased oligomerization (Fig. 6, *B* and *C*) but was strongly loss of function in the permeable cell assay (Fig. 6*E*). By contrast, a CAPS(G476A) variant was similar to wild-type protein (data not shown). Collectively, the data indicate that the C2 domain of CAPS, previously of unknown function, is involved in CAPS dimer formation and that altered dimerization inhibits CAPS function.

##### C2 Domain Is Essential for CAPS Function in Cells

**FIGURE 7.**
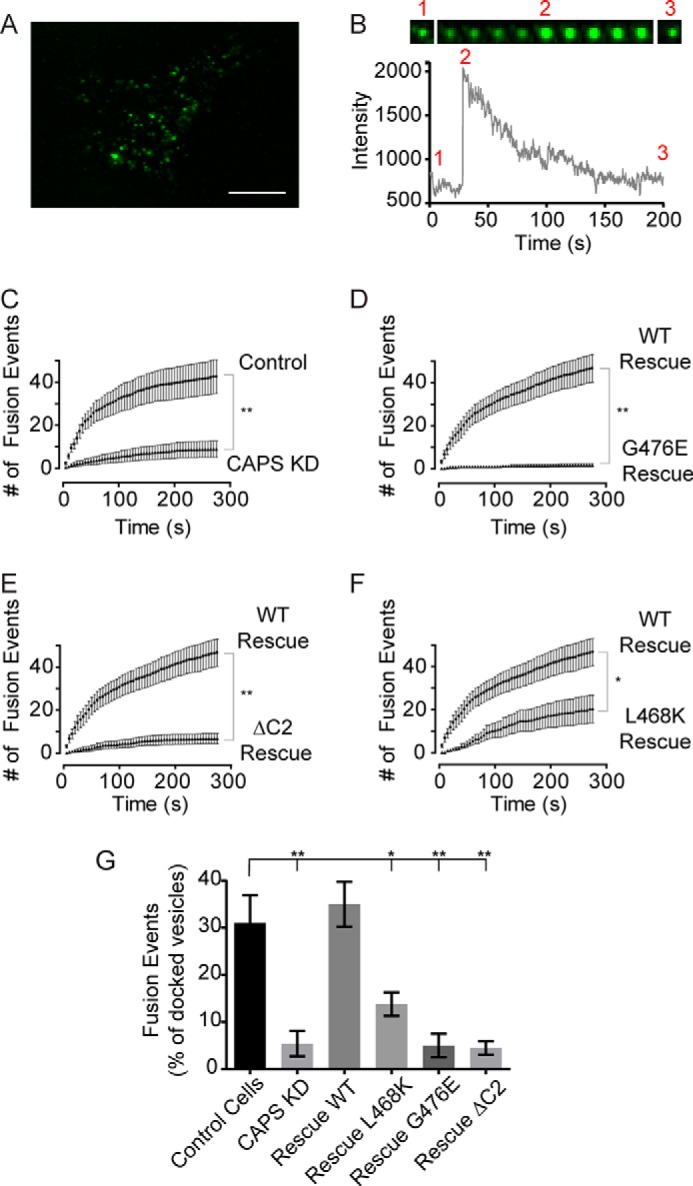
**C2 domain mutants exhibit reduced activity in dense core vesicle exocytosis.** *A*, representative TIRF image of PC12 cell expressing BDNF-GFP. *Scale bar*, 10 μm. *B*, fluorescence intensity changes for a single vesicle fusion event. Vesicle fluorescence is evident in the TIRF field before fusion (*1*); fluorescence increases at the time of fusion pore opening (*2*); and fluorescence subsequently decreases due to reacidification of the cavicaptured vesicle (*3*). The width of each image in the montage is 1.5 μm. *C*, exocytosis was evoked by depolarization with the addition of 56 mm KCl buffer at zero time. Curves indicate cumulative number of fusion events per cell. CAPS knockdown significantly impaired secretion compared with control cells (mean ± S.E. (*error bars*), *n* = 14). *D*, rescue studies in CAPS knockdown cells for CAPS-TagRFP (*n* = 14) or CAPS(G476E)-TagRFP (*n* = 11). *E*, similar to *D* for rescue studies with CAPS-TagRFP (*n* = 14) or CAPS(ΔC2)-TagRFP (*n* = 15). *F*, similar to *D* for rescue studies with CAPS-TagRFP (*n* = 14) or CAPS(L468K)-TagRFP (*n* = 15). The similar expression of CAPS proteins was confirmed by fluorescence of CAPS-TagRFP constructs. Significant differences are indicated: **, *p* < 0.001; *, *p* < 0.05. *G*, summary of CAPS C2 domain mutants compared with wild-type CAPS. Fusion events per cell were normalized to the number of vesicles visible in the TIRF field (mean ± S.E.; *, *p* < 0.05; **, *p* < 0.001).

**FIGURE 8.**
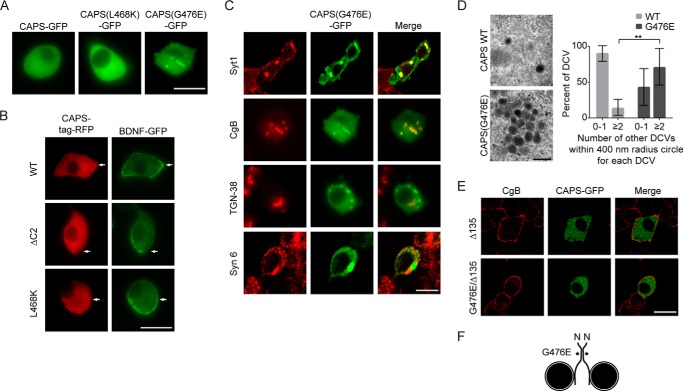
**Localization of CAPS C2 domain mutants in PC12 cells.** *A*, representative confocal images of PC12 cells expressing wild-type or mutant CAPS-GFP fusion proteins. Wild-type CAPS and CAPS(L468K) are broadly distributed in the cytoplasm, whereas CAPS(G476E)-GFP localizes to large structures. *Scale bar*, 10 μm. *B*, confocal images of live PC12 cells expressing BDNF-GFP (vesicle cargo) and either wild-type CAPS, CAPS(ΔC2), or CAPS(L468K) as TagRFP fusions. *Scale bar*, 10 μm. *C*, confocal images of fixed PC12 cells expressing CAPS(G476E)-GFP and immunostained for dense core vesicle proteins synaptotagmin-1 (*Syt1*) or chromogranin B (*CgB*), for *trans*-Golgi protein TGN-38, or for Golgi and immature vesicle protein syntaxin-6 (*Syn-6*). *Scale bar*, 15 μm. *D*, electron micrograph of fixed PC12 cell expressing CAPS-GFP or CAPS(G476E)-GFP. An *enlarged region* containing clusters of dense core vesicles is shown. *Scale bar*, 200 nm. Clustering was determined by the number of additional vesicles in a 400-nm radius surrounding a vesicle; binning results for 0–1 or ≥2 vesicles/circle are shown. CAPS(G476E)-GFP (*n* = 7 sections) significantly increased the percentage of vesicles with ≥2 adjacent vesicles (**, *p* < 0.001) compared with CAPS-GFP (*n* = 8 sections). *Error bars* indicate S.D. *E*, CAPS(G476E) clustering of dense core vesicles is dependent on the vesicle binding domain of CAPS. C-terminal truncation of 135 residues from CAPS abolished vesicle clustering by CAPS(G476E). *Scale bar*, 15 μm. *F*, model of dense core vesicle clustering by CAPS(G476E) depicting C2 domain self-interactions and C-terminal vesicle-binding domain.

We assessed the activity of the C2 domain mutants in regulated dense core vesicle exocytosis in live PC12 cells (19). Cells expressing brain-derived neurotrophic factor (BDNF)-EGFP as vesicle cargo (Fig. 7*A*) were depolarized to promote Ca^2+^ influx. Vesicle fusion events were detected by the brightening of BDNF-EGFP in TIRF microscopy (Fig. 7*B*). The knockdown of CAPS in PC12 cells with shRNA (>95% reduction in CAPS (19)) strongly reduced Ca^2+^-stimulated exocytosis (Fig. 7*C*). Full rescue was achieved by expressing a CAPS-TagRFP construct harboring silent mutations that bypass the shRNA (Fig. 7*D*). By contrast, CAPS(G476E), CAPS(ΔC2), or CAPS(L468K) proteins all failed to rescue Ca^2+^-stimulated exocytosis (Fig. 7, *D–F*) despite normal expression (Fig. 8). Intriguingly, CAPS(G476E) expression also strongly reduced the number of dense core vesicles visible in the TIRF field, whereas expression of other mutants did not (data not shown). To determine whether reduced exocytosis with CAPS(G476E) expression (Fig. 7*D*) was solely attributable to the reduced number of vesicles in the TIRF field, the data were replotted as the number of fusion events normalized to the number of vesicles in the TIRF field. CAPS(G476E) was confirmed as strong loss of function for exocytosis as were the CAPS(L468K) and ΔC2 CAPS mutants (Fig. 7*G*). The live cell studies agree with the permeable cell activity assay results (Fig. 6*E*) and further indicate the essential nature of the C2 domain for CAPS function in vesicle exocytosis.

##### CAPS(G476E) Cluster Dense Core Vesicles

CAPS in PC12 cells is cytoplasmic as well as bound to dense core vesicles (3). CAPS(G476E) was loss of function for vesicle exocytosis (Fig. 7, *D* and *G*) but also interfered with the transit of vesicles to the plasma membrane. To further assess this, we determined the localization of CAPS(G476E) in cells by confocal microscopy. Expression of wild-type CAPS-EGFP or CAPS-TagRFP resulted in a global cytoplasmic distribution that largely obscured the dense core vesicle localization previously shown by TIRF microscopy (3) (Fig. 8, *A* and *B*). CAPS(L468K) and CAPS(ΔC2) proteins were expressed well and localized similarly to the wild-type protein as cytoplasmic. In striking contrast, CAPS(G476E) localized to large structures within the cells (Fig. 8, *A* and *C*) that also contained dense core vesicle proteins synaptotagmin-1 and chromogranin B but not Golgi proteins TGN-38 or syntaxin-6 (Fig. 8*C*). This suggested that CAPS(G476E) expression induced the cytoplasmic clustering of mature dense core vesicles. We confirmed this by electron microscopy, which revealed cytoplasmic clusters of intact dense core vesicles in cells expressing CAPS(G476E) (Fig. 8*D*).

Vesicle clustering by CAPS(G476E) may be due to its increased oligomerization (Fig. 6*B*) coupled with its binding to dense core vesicles (Fig. 8*F*). CAPS binding to dense core vesicles requires a C-terminal domain (Fig. 6*A*) (3, 25). We constructed a C-terminally truncated version of CAPS(G476E) and found that vesicle clustering was eliminated (Fig. 8*E*). The results indicate that the G476E mutation enhances CAPS self-interactions even in *trans* across vesicle membranes. Such results are consistent with a role for the C2 domain in CAPS dimerization (Fig. 8*F*).

##### Bioinformatic Analysis of CAPS C2 Domain

Dimerization may be mediated by homotypic interactions between C2 domains. Structures of C2 domain homodimers have been reported for C2B of RIM1α, C2B of Rgp3, and C2A of Munc13-1 (37, 38). Analysis of these structures reveals several conserved patterns for C2 homodimer formation. Residues involved in dimerization, while uniquely positioned on each structure, are in the same β-strands. These residues interact with residues on other β strands or with loops that connect the β strands. Each of the three structures contains an electrostatic lysine-aspartate interaction stabilized by interaction with an aromatic residue and by a hydrogen bond from a third residue (see below). We determined whether any of these dimerization residues were conserved in CAPS by conducting C2 domain alignments with Cn3D, which supplements traditional sequence alignment methods with structural data (39).

**FIGURE 9.**
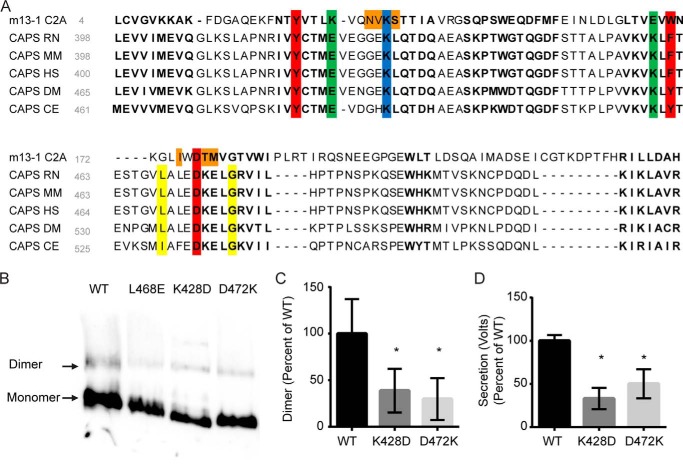
**Identifying conserved homodimerization motifs in Munc13-1 C2A and CAPS C2.** *A*, sequence alignment of C2 domains generated by Cn3D. A spatially conserved residue alignment for C2 domains was created with 97 published C2 structures. This template was used to compare Munc13-1 C2A (Protein Data Bank code 2CJT), CAPS RN (*R. norvegicus*, NP_037351), CAPS MM (*Mus musculus*, NP_036191), CAPS HS (*Homo sapiens*, Q9ULU8), CAPS DM (*Drosophila melanogaster*, AAN06591), and CAPS CE (*C. elegans*, NP_001255666) sequences. *Boldface type* indicates residues that are spatially conserved across all 97 structures. *Red* and *blue*, partial chelation of a charged residue (lysine, *blue*) by 3 opposing residues (*red*) is conserved for Munc13-1 and CAPS. *Green*, electrostatic interaction between a lysine and glutamine is conserved spatially, although the residue positions are inverted between CAPS and Munc13-1. *Orange*, Munc13-1 homodimer interactions that involve residues with variable spatial positioning between C2 domains. *Yellow*, Leu-468 and Gly-476 residues identified in CAPS (22), where mutation causes loss of function. *B*, charge reversal mutations (K428D and D472K) in residues conserved in Munc13-1 C2A and CAPS C2 domains impair CAPS dimerization. Similar amounts of the indicated proteins were analyzed by CN-PAGE and Western blotting with CAPS antibody. *C*, data from *B* were quantitated, indicating decreased dimerization in K428D and D472K proteins compared with wild type (*, *p* < 0.05, mean ± S.D. (*error bars*), *n* = 3). *D*, CAPS(K428D) and CAPS(D472K) proteins were tested at 20 nm in the permeable cell secretion assay and found to be significantly impaired compared with wild-type CAPS (*, *p* < 0.05, mean ± S.D., *n* = 3).

Alignments revealed that residues involved in Munc13-1 C2A homodimerization are conserved in the CAPS C2 domain (Fig. 9*A*). Specifically, two types of interactions are conserved. Both C2 domains exhibit a characteristic partial chelation of a charged residue by three residues on the opposing domain and exhibit a lysine-glutamate electrostatic interaction (Fig. 9*A*). The positions of these latter residues are flipped between CAPS C2 and Munc13-1 C2A, which could function to prevent CAPS-Munc13-1 heterodimer formation. To test the sequence alignment predictions, we generated two charge reversal mutants in the CAPS C2 domain that are predicted to reduce dimerization. In accord with prediction, CAPS(K428D) and CAPS(D472K) exhibited impaired dimerization (Fig. 9, *B* and *C*). Importantly, both CAPS C2 domain mutants showed reduced activity in the permeable PC12 cell assay (Fig. 9*D*). We suggest that the mechanism of C2 domain homodimerization is conserved between the CAPS C2 domain and the Munc13-1 C2A domain.

## Discussion

### 

#### 

##### Essential Role of CAPS C2 Domain

We provide evidence that the C2 domain is essential for CAPS activity as a regulator of vesicle exocytosis. This conclusion initially emerged from sequencing the *unc-31(e714*) *and unc-31(ox299*) loss of function alleles in *C. elegans* that correspond to rat CAPS(G476E) and CAPS(L468K), respectively (22). These single point mutations in the CAPS C2 domain are separated by seven residues. Studies of regulated dense core vesicle exocytosis (Figs. 6 and 7) showed that CAPS(G476E) was entirely inactive, whereas CAPS(L468K) was partially active. Importantly, CAPS(ΔC2) exhibited no activity (Figs. 6 and 7). The results strongly indicate that the C2 domain is required for CAPS activity in Ca^2+^-dependent dense core vesicle exocytosis.

The CAPS C2 domain lacks the canonical array of five aspartate residues characteristic of Ca^2+^-dependent, PS-binding C2 domains (40). We were unable to demonstrate Ca^2+^-dependent PS binding for CAPS C2 domain constructs that we have produced. However, C2 domains in proteins also mediate protein-protein interactions (38, 41–47). C2 domains can also homodimerize, such as in piccolo, dysferlin, RIM1a, Munc13-1, and Rgp3 (38, 48–50). That the CAPS C2 domain mediates homodimerization is suggested by the findings that monomeric CAPS in solution is in dynamic equilibrium with a pool of dimer and that a large number of C2 domain mutant CAPS proteins (L468E, L468A, L468K, K428D, D472K, and ΔC2) exhibit strong reductions in dimerization. It is unlikely that these mutant CAPS proteins are either globally or locally misfolded because they express at wild-type levels in cells, bind PI(4,5)P_2_ similarly to wild-type CAPS, and exhibit similar patterns of limited proteolysis.^4^ It is likely that these mutant proteins exhibit reduced affinity for C2 domain self-interactions.

The CAPS(G476E) mutant corresponding to the *unc-31(e714)* allele has unusual properties that nonetheless support the idea of C2 domain-mediated oligomerization. CAPS(G476E) is strongly loss of function at the level of vesicle exocytosis (Figs. 6 and 7), and it also clusters dense core vesicles in the cytoplasm of cells (Fig. 8). We interpret this to indicate that the G476E C2 domain gains affinity for self-interaction so that it dimerizes in *trans* across membranes (Fig. 8*F*). Indeed, CAPS(G476E) exhibits a very strong tendency to oligomerize as dimers as well as possible tetramers (Fig. 6*B*). We cannot eliminate the possibility that the C2 domain in this mutant is locally misfolded by substitution of a small uncharged side chain by a charged side chain. The G476A allele, by contrast, exhibited wild-type properties. Gly-476 is highly conserved in CAPS and in other C2 domains (Fig. 9*A*), residing near the beginning of a flexible linker that connects two β strands. Whether the Gly to Glu substitution promotes local unfolding of the C2 domain or introduces additional electrostatic interactions is unclear, but the G476E substitution appears to strongly enhance C2-C2 domain interactions.

That both decreased (*e.g.* CAPS(ΔC2) and CAPS(L468K)) as well as increased (*e.g.* CAPS(G476E)) dimerization result in loss of CAPS function suggests that a cycle of dimerization and its reversal plays a role in CAPS function. CAPS dimerization may need to be locally activated with correct timing such that premature dimerization inhibits activity. Consistent with this, complete dephosphorylation inactivates CAPS and shifts it toward an oligomeric state (31). It should be noted that the current work reassigns the speculated dimer-tetramer equilibrium of that previous study (31) to a monomer-dimer equilibrium.

##### Distinct Roles for Dimerization in CAPS and Munc13-1 Function

CAPS and Munc13-1 proteins comprise a subgroup of CATCHR family proteins exhibiting ∼40% sequence similarity in a ∼500-residue region that is C-terminal to the C2 domain of CAPS. Dimerization of the Munc13-1 protein has not been directly determined but is proposed based on C2A-C2A dimer structures (38). Mutations that disrupt C2A-C2A dimers (K32E) were found to enhance Munc13-1 function in neurons, suggesting that Munc13-1 monomers are active (50). A proposed mechanism for Munc13-1 activation involves RIM1-induced dissociation of C2A-C2A dimers to activate Munc13-1 as a monomer (38, 50). This contrasts with our results for CAPS, which indicate that C2 domain-mediated dimerization is required for function in vesicle exocytosis. The monomer-dimer equilibrium strongly favoring monomer for CAPS indicates that the affinity for self-interaction between soluble CAPS monomers is low, which contrasts with the reported high affinity for Munc13-1 C2A-C2A interactions (38).

Although CAPS and Munc13-1 are each proposed to promote the assembly of SNARE complexes for vesicle priming, the two proteins function distinctively and non-redundantly (3, 7, 12, 19, 21). CAPS promotes the formation of dimers of heterotrimeric SNARE complexes on liposomes, which may be mediated by multivalent interactions of an active membrane dimer of CAPS with SNARE proteins (30). This is similar to mechanisms suggested for a number of dimeric tethering factors (51, 52). By contrast, Munc13-1 is suggested to open a closed form of syntaxin-1 (53), which may be well suited for an active monomeric Munc13-1 protein.

Despite divergent roles for dimerization in function, the mechanism of dimerization for CAPS and Munc13-1 may be similar. Munc13-1 C2A and CAPS C2 domains exhibit 41% amino acid sequence similarity, and neither is a Ca^2+^-dependent phospholipid-binding domain (50). Conserved residues in the CAPS C2 domain (Lys-428 and Asp-472) were identified based on the Munc13-1 C2A homodimer structure (38). Charge reversal mutations in these residues impaired CAPS dimerization and function, supporting the conclusion that dimerization mechanisms for CAPS and Munc13-1 are conserved.

##### Regulation of CAPS Dimer Formation and Function

The binding of PI(4,5)P_2_ to CAPS markedly enhanced dimer formation (Fig. 3). Binding of PI(4,5)P_2_ to the PH domain of CAPS (25) may induce dimer formation by synergistic interactions within the central C2-PH region of CAPS (Fig. 6*A*). The PH domain is immediately adjacent to the C2 domain so that binding of PI(4,5)P_2_ in the membrane could drive C2 domain-mediated dimerization. PH domains can also dimerize (54, 55), which could account for the incomplete loss of dimerization in CAPS(ΔC2). Dimerization of the PH domain in CAPS could increase the avidity for binding PI(4,5)P_2_, which would further stabilize dimers on the membrane. More C-terminal domains in dimerized CAPS such as MHD1 might in turn exhibit multivalent interactions with SNARE proteins to promote the assembly of dimers of SNARE complexes, as observed on liposomes (30). Thus, the unique central C2-PH domain of CAPS could serve as an important PI(4,5)P_2_-dependent switch for the activation of CAPS as a dimer on membrane. This model would account for the essential roles for PI(4,5)P_2_ binding and dimerization in CAPS function.

## Experimental Procedures

### 

#### 

##### DNA Constructs

A site-directed mutagenesis kit (Qiagen, Hilden, Germany) was used with pcDNA3.1 CAPS-Myc-His (*Rattus norvegicus*) as the template to introduce L468A, L468E, L468K, G476A, G476E, K428D, or D472K single point mutations. For the ΔC2-CAPS, overlap extension PCR was used to delete amino acids 400–516 from the CAPS ORF. This allowed residue 399 to be directly fused to residue 517 without introduction of a restriction site. Primers used were f1 (CCAGGACCGATTCCAGGCTTTCCTCAATGG, f2 (TCCTTCTCCTTGGAGCAAAACATGAAGCACTCTGG), r1 (AGAGTGCTTCATGTTTTGCTCCAAGGAGAAGGACAG), and r2 (CCTGAAATGTGTAATTTGATTTTCCAGCAGAACTCGG). CAPS-mKATE2-His constructs were created by cutting CAPS-Myc-His from pcDNA3.1 with XhoI and AfeI and ligating into a similarly cut mKate2-C (Evrogen, Moscow, Russia) appended with a C-terminal His purification tag. All constructs were confirmed by Sanger sequencing.

##### Protein Purification

For purification of CAPS-Myc-His and CAPS-mKate2-His, CAPS was transfected into HEK293-FT (Invitrogen) cells using a standard calcium phosphate protocol. Two days after transfection, cells were washed and removed with PBS. Cells were resuspended in 0.5% Triton X-100, 20 mm HEPES, 300 mm NaCl, 5 mm imidazole (IDA) with protease inhibitors and pelleted to remove cell debris. Supernatants were loaded onto a nickel-nitrilotriacetic acid column (Qiagen), washed, and eluted with 20 mm HEPES, 300 mm NaCl, 200 mm IDA. Eluates were diluted with 20 mm HEPES to 20 mm NaCl, 100 mm NaCl and concentrated to ∼10 μm CAPS.

##### Blue and Clear Native PAGE

For clear native PAGE, purified CAPS-Myc-His and mutants in 20 mm HEPES, 10 mm NaCl, 10 mm imidazole, 5% glycerol, pH 7.4, were loaded onto a 6% polyacrylamide gel without SDS or DTT. Gels were run at 150 V for 2 h at 4 ºC. For immunodetection, native gels were subjected to a standard blotting protocol. Blue native PAGE was performed as described previously (56). Briefly, 5–14% acrylamide resolving gels were poured with a 3.3% acrylamide stacking gel (Buchler Instruments, Fort Lee, NJ). Protein samples were mixed with 2× sample buffer (0.25% Coomassie G250, 75 mm BisTris, 10% glycerol, 1.5 m 6-aminocaproic acid, pH 7.0) and directly loaded into the gel. Gels were run at 150 V through the stack and then turned up to 250 V for 2 h. Gels were fixed in 40% methanol, 30% acetic acid, 2.5% glycerol followed by Coomassie G250 staining or subjected to immunoblotting. Western blotting was conducted with polyclonal antibodies generated against purified full-length rat CAPS or with Myc monoclonal 9E11 (Covance, Dedham, MA).

##### Analytical Ultracentrifugation

Three different CAPS concentrations (0.2–8.4 μm) were analyzed. ∼100 μl of each sample was loaded into sectors of 1.2-cm charcoal-filled epon centerpieces with ∼110 μl of the final dialysate buffer in the reference sector. Data were collected at 4 °C with the gradients monitored at 279 nm. From the CAPS sequence, the partial specific volume was 0.733 ml/g and is uncorrected for temperature. The buffer composition was 100 mm NaCl, 1 mm EDTA in HEPES buffer at pH 7.4. The density at 4 °C was 1.0044 g/ml, as calculated from density increments (Laue diffraction) and corrected to 4 (no increment was available for HEPES, and its contribution was neglected). CAPS molecular mass was computed as 150,613. An extinction coefficient of 138,660/m/cm was used based on Pace's average extinction (58) and does not include the disulfide bonds. Equilibrium data were collected at 3600, 6000, and 8800 rpm. Equilibrium was assumed when gradients collected ≥3 h apart were superimposable. After a high speed spin for a few hours depleted all protein material, the remaining absorbance in the low field region was taken as a measure of non-sedimentable absorbance, which was <0.01 and treated as fixed during various model fittings. The range of absorbance in the data included in the analysis ranged from 0.03 to 1.4 (at 1.2-cm path length) or a concentration of 0.2–8.4 μm. For data shown (Fig. 1*D*), the *three lines* for each *color* represent different rotor speeds for the high (*green*), medium (*blue*), and low (*red*) loading concentration.

##### Atomic Force Microscopy

CAPS was imaged by adding 100 pm CAPS to freshly cleaved mica disks and allowed to dry or imaged submerged with SLBs at 10 nm. 1,2-Dioleoyl-*sn*-glycero-3-phosphocholine (DOPC), 1,2-dioleoyl-*sn*-glycero-3-phospho-l-serine (DOPS), and 1,2-dioleoyl-sn-glycero-3-phospho-(1′-myo-inositol-4,5-bisphosphate) (PI(4,5)P_2_), obtained from Avanti Polar Lipids (Birmingham, AL) as chloroform stocks, were mixed at molar ratios of 75% phosphatidylcholine, 25% phosphatidylserine or 75% phosphatidylcholine, 22% phosphatidylserine, 3% PI(4,5)P_2_. Chloroform was evaporated under a stream of nitrogen, and the lipids were rehydrated overnight in water (MilliQ) at a lipid concentration of 2 mg/ml. The lipid suspension was incubated at 65 °C for 30 min and then cooled to room temperature. CAPS, NaCl, HEPES-NaOH, and CaCl_2_ were added to produce a liposome suspension containing 10 nm CAPS in HEPES-buffered saline (HBS; 100 mm NaCl, 20 mm HEPES, pH 7.6) and 1 mm CaCl_2_. The suspension was incubated at 37 °C for 30 min and then deposited onto freshly cleaved mica disks (Agar Scientific, Stansted, UK). After a 3-min adsorption, the sample was rinsed with HBS containing CaCl_2_ to remove unadsorbed liposomes and transferred to the atomic force microscope. AFM imaging was carried out at room temperature using a Bruker Multimode atomic force microscope equipped with an E-scanner and a Nanoscope IIIa controller, with an in-line electronics extender module (Bruker, Coventry, UK). All images were collected using tapping mode in fluid with Micromasch AFM cantilevers NSC18/AL BS MikroMasch® (Innovative Solutions Bulgaria Ltd., Sofia, Bulgaria). The cantilevers (typically exhibiting a spring constant of 2.8 newtons/m) were tuned to 10–20% below the peak of the resonance frequency, generally between 25 and 35 kHz. The drive amplitude was set to generate a root mean square amplitude of 2 V. The microscope was engaged with a 0-nm scan area to allow for tuning. The set point was applied to the sample. Images (2 × 2 μm) were captured at a scan rate of 2 Hz and with 512 scan lines/area. Data analysis was performed using commercially available software (NanoScope III, Digital Instruments) or with ImageJ. Multiple images were taken from one supported lipid bilayer for each condition.

##### Single Molecule Microscopy

Single molecules of CAPS-mKate2-His, mKate2-His, or the dimeric (mKate2)_2_-His were immobilized on glass by SiMPull (35). Briefly, an imaging chamber was created by sandwiching 24 × 60-mm number 1.5 and 22 × 60-mm number 1.5 glass slides between lines of high vacuum grease to create 4–5 channels with a volume between 20 and 50 μl. Chambers were rinsed with 10 chamber volumes of buffer (25 mm HEPES, 100 mm KCl, pH 7.4) to remove dust and a BSA/antibody solution (FC protein (10 mg/ml), monoclonal His tag antibody (Penta-His (Qiagen)) concentration of 0.1 mg/ml), incubated for 10 min, and flushed with 5 chamber volumes of buffer, and 50 pm CAPS-mKate2-His was added to the chamber; protein was diluted in prelubricated 1.7-ml Eppendorf tubes to minimize nonspecific absorption to the plastic surface (Costar, catalog no. 3207). Alternatively, CAPS-mKate2-His was immobilized on 5% PI(4,5)P_2_-containing SLBs. CAPS-mKate2 lacking the His tag was prepared by cleavage with tobacco etch virus protease (R&D Systems, Minneapolis, MN) and similarly bound to SLBs. Unbound protein was removed with 5 chamber volumes of buffer, and the bound protein was imaged by TIRF microscopy on a TE2000 inverted scope (Nikon) stationed on a floating table. A 514-nm laser line excited the fluorophore and was imaged with an Evolve digital monochrome EMCCD camera (Photometrics, Tucson, AZ). 500-frame movies with a capture rate of 2 Hz were recorded. Movies were drift-corrected using the ImageJ plugin Descriptor-based registration (2d/3d +t); movies with significant drift were discarded. Movies were processed via a Matlab script generously provided by Dr. Aaron Hoskins (University of Wisconsin, Madison, WI). Photobleaching steps were manually recorded if a spot maintained uniform fluorescence before photobleaching, did not display *x*,*y*-drift, and underwent photobleaching in one frame.

##### Gel Filtration

Gel filtration of purified CAPS-Myc-His was conducted on a Sephacryl-300 (Amersham Biosciences, Little Chalfont, UK) prepacked column via FPLC. The column was calibrated by monitoring the elutions of thyroglobulin (670 kDa), ferritin (440 kDa), and aldolase (158 kDa). Purified CAPS-Myc-His was loaded onto the column, and 72 1.2-ml fractions were collected and analyzed by clear native gels and immunoblotting.

##### Cell Ghost Binding

PC12 cells were washed twice with PBS, removed with KGlu/BSA buffer (120 mm potassium glutamate, 20 mm potassium acetate, 20 mm HEPES, 2 mm EGTA, 1 mg/ml BSA, pH 7.2), and passed once through a ball homogenizer (10-μm clearance). 100 mm EGTA was added to a final concentration of 10 mm, and permeable cells were incubated on ice for 30–60 min and washed with KGlu/BSA to remove cytosolic proteins. Cell ghosts were divided into Eppendorf tubes, resuspended in 20 nm CAPS-Myc-His in priming solution (Mg-ATP/rat brain cytosol) for 12 min at 30 °C, and washed two times in 1 ml of KGlu/BSA. Bound CAPS was either extracted with 300 mm NaCl (high salt) or loaded directly onto native gels. 50% glycerol was added to a final concentration of 5% before loading onto native gels.

##### Liposome-binding Assays

Proteoliposomes were prepared by co-micellization as described previously (23). Briefly, syntaxin-1/SNAP-25 in 25 mm HEPES, pH 7.4, 100 mm KCl, 50 mm IDA, 1% β-octyl glucoside was used to resuspend a dried film of 2.5 mm lipid of DOPC, 5% PI(4,5)P_2_. After resuspension, mixtures were rapidly diluted into 25 mm HEPES, 100 mm KCl, pH 7.4; dialyzed overnight into 25 mm HEPES, 100 mm KCl, 1 mm DTT, pH 7.4, with 1 g/liter Biobeads; and purified on an Accudenz medium (Accurate Chemical Corp., Westbury, NY) gradient. Liposomes were made identically with the omission of the SNARE proteins. The addition of ^3^H-labeled 1,2-dipalmitoyl phosphatidylcholine to liposomes was used to monitor lipid recovery, and SDS-PAGE densitometry of the Q-SNAREs was used to assess protein content. 1 μm CAPS-Myc-His was incubated with Q-SNARE proteoliposomes for 30 min at room temperature. Reactions were mixed 1:1 with 80% Accudenz and pipetted into a thin walled polycarbonate tube, overlaid with 30% Accudenz and a 20 mm HEPES, 100 mm KCl, pH 7.4, layer for centrifugation (45000 rpm, SW50 swinging bucket rotor). Floated fractions were withdrawn and analyzed by either blue native or SDS-PAGE. For blue native analysis where indicated, Triton X-100 was added to a final concentration of 0.5% for several minutes followed by 2× blue native PAGE buffer (0.25% Coomassie G250, 75 mm BisTris, 10% glycerol, 1.5 m 6-aminocaproic acid, pH 7.0) for 5 min. When quantification was required, floated fractions were monitored by the total amount of ^3^H-labeled 1,2-dipalmitoyl phosphatidylcholine.

##### Permeable Cell Secretion Assay

Rotating disc electrode voltammetry was used to detect norepinephrine secretion from PC12 cell ghosts as described previously (4) using PC12 cells loaded overnight with 1.5 mm norepinephrine, 0.5 mm ascorbate. Cell ghosts were prepared as described above, washed in KGlu/BSA, and incubated in priming solution (rat brain cytosol at 1:10 dilution and 2 mm Mg-ATP) for 12 min at 30 °C, which generates PI(4,5)P_2_ on the plasma membrane, washed, and resuspended in KGlu/BSA. Cell ghosts were added directly to the rotating disc electrode chamber with 10 nm CAPS, and Ca^2+^ was injected. A rotating carbon disc electrode with an applied potential of +500 MV detected norepinephrine release. The output was processed by a Dataq analog to digital data acquisition system and analyzed by WinDaq software.

##### Live PC12 Secretion Assay

The Ca^2+^-dependent exocytosis of BDNF-GFP-containing vesicles from live PC12 cells was monitored by TIRF microscopy as described previously (19). Briefly, PC12 cells were transfected with BDNF-GFP, a CAPS shRNA plasmid, and a CAPS-mKate2 construct and plated on MatTek glass bottom dishes. After 48 h, cells were imaged by TIRF on a Nikon TE2000-U microscope. Resting cells in 15 mm HEPES, pH 7.4, 145 mm NaCl, 5.6 mm KCl, 2.2 mm CaCl_2_, 0.5 mm MgCl_2_, 5.6 mm glucose, 0.5 mm ascorbic acid, 0.1% BSA were stimulated by buffer exchange into 15 mm HEPES, pH 7.4, 95 mm NaCl, 56 mm KCl, 2.2 mm CaCl_2_, 0.5 mm MgCl_2_, 5.6 mm glucose, 0.5 mm ascorbic acid, 0.1% BSA. GFP fluorescence was excited with a 488-nm laser, and mKate2 fluorescence was excited with a 514-nm laser and imaged at 4 Hz with either a CoolSNAP-ES digital monochrome CCD camera (Photometrics, Tucson, AZ) or an Evolve digital monochrome EMCCD camera (Photometrics) and analyzed by ImageJ software. Exocytic events were scored manually.

##### Confocal and Electron Microscopy

PC12 cells were transfected with an Electroporator II instrument (Invitrogen) and plated on glass coverslips, and 48 h later, cells were fixed with 4% formaldehyde plus PBS for 20 min at room temperature. For immunostaining, fixed cells were extracted in 0.1% Triton X-100. Cells were washed with PBS and blocked for 30 min in 10% BSA plus PBS. Fixed cells were incubated with primary antibody for 1 h, washed, and incubated with secondary antibody for 1 h. After washes in PBS, cells were mounted and imaged on a Nikon C1 confocal microscope (TE2000-U). Primary antibodies used were chromogranin B monoclonal generously provided by W. B. Huttner (Max Planck Institute of Molecular Cell Biology and Genetics, Dresden, Germany), TGN38 monoclonal generously provided by K. E. Howell (University of Colorado, Denver, CO), syntaxin 6 monoclonal antibody (catalog no. 610636, BD Biosciences), and synaptotagmin-1 N-terminal polyclonal antibody (Sigma-Aldrich). For electron microscopy, PC12 cells were transfected with pCMV CAPS-GFP or CAPS(G476E)-GFP for 48 h, sorted by FACS on a SORP BD FACSAria (University of Wisconsin Carbone Cancer Center Flow Cytometry Laboratory) to remove untransfected cells, and fixed. Cells on glass coverslips were fixed in a solution of 2.5% glutaraldehyde, 2.0% paraformaldehyde in 0.1 m sodium phosphate buffer (PB), pH 7.4, for ∼1 h at room temperature. Samples were rinsed 5 times for 5 min in 0.1 m PB. The rinsed cultures were post-fixed in 1% osmium tetroxide, 1% potassium ferrocyanide in PB for 1 h at room temperature. The samples were rinsed in PB followed by distilled water rinses three times for 5 min each to clear phosphates. Samples were en bloc stained in saturated aqueous uranyl acetate for 2 h at room temperature and rinsed in distilled water three times for 5 min each. Dehydration was performed at room temperature in a graded ethanol series (35, 50, 70, 80, and 90% for 5 min, 95% for 10 min, 100% three times for 10 min) and transitioned in propylene oxide two times for 7 min each. Durcupan ACM (Fluka AG, Buchs, Switzerland) resin was used during infiltration and embedding. Increasing concentrations of accelerated (10 ml of A/M, 10 ml of B, 300 μl of C, 100 μl of D components) Durcupan were used for infiltration. All infiltration steps were done with the coverslips, cell side up, in aluminum weighing dishes in covered glass Petri dishes to minimize evaporation. The cultures were embedded in open aluminum weighing dishes at 60 °C in a drying oven overnight until polymerized. Samples were floated in concentrated hydrofluoric acid, glass side down, for 15 min to etch off the glass, revealing the embedded cells. Sections were viewed with a Philips CM120 transmission electron microscope, and images were documented with a MegaView III (Olympus-SIS, Lakewood, CO) side-mounted digital camera.

##### Sequence Alignments

We used the C2 domain conserved protein domain family (CD00030) as a seed for C2 domain alignments. It is produced by an automated algorithm, which employs generic search parameters that invariably contain omissions and misalignments that require refinement. Only 32 of 97 C2 domain structures represented in the C2 domain PFAM (PF00168) were included (at the time of submission, this PFAM has 105 structures). The missing structures were imported into Cn3D and aligned with the provided block alignment algorithm. The sequence alignment of every structure was manually refined by assessing the resulting spatial overlap between the C2 domain in question with all other C2 structures. After assessing every β strand for all structures, we were able to create a sequence alignment for all C2 domain structures that is based on a high degree of spatial overlap between all C2 domain residues. The aligned residues represent the core C2 domain residues that have an invariant spatial positioning between these 97 crystal structures. Using these core C2 residues as a search query, DALI identified all other C2 domain structures in the C2 PFAM, yielding an average root mean square deviation of 1 Å that accounts for 100 of the residues tested for spatial overlap. It is common to observe backbone atoms of two aligned C2 domains in these conserved regions with distances between 0.4 and 0.6 Å, less than that of an H–H bond.

## Author Contributions

M. P., J. E., and T. F. J. M. designed the study and wrote the paper. M. P. conducted most of the experimental work; J. E. conducted single molecule, bioinformatic, and mutagenesis studies; G. K. conducted cellular studies on CAPS mutants; and S. M. conducted permeable cell secretion assays. H. T. and J. M. E. designed and conducted the AFM studies. All authors reviewed the results and approved the final version of the manuscript.

## Footnotes

4 M. Petrie, unpublished observations.

## Footnotes

3 The abbreviations used are:

CAPS: Ca^2+^-dependent activator protein in secretion
PI(4,5)P_2_: phosphatidylinositol 4,5-bisphosphate
C2: domain homologous to second domain of protein kinase C
PH: pleckstrin homology
CATCHR: complexes associated with tethering containing helical rods
MHD: Munc13 homology domain
TIRF: total internal reflection fluorescence
SLB: supported lipid bilayer
AFM: atomic force microscopy
IDA: imidazole
BisTris: 2-[bis(2-hydroxyethyl)amino]-2-(hydroxymethyl)propane-1,3-diol
DOPC: 1,2-dioleoyl-*sn*-glycero-3-phosphocholine
DOPS: 1,2-dioleoyl-*sn*-glycero-3-phospho-l-serine
PC: phosphatidylcholine
PS: phosphatidylserine
CN: clear native gel
BN: blue native gel.
